# First record of *Argulus japonicus* infestation on *Cyprinus carpio* in Hungary, and the first description of *Argulus japonicus* *europaeus* subsp. nov. Keve, 2025

**DOI:** 10.1186/s13071-025-07106-1

**Published:** 2025-11-21

**Authors:** Gergő Keve, Adrienn Gréta Tóth, Máté Katics, Ferenc Baska, Edit Eszterbauer, Sándor Hornok, Tibor Németh, Norbert Solymosi

**Affiliations:** 1 Department of Parasitology and Zoology, University of Veterinary Medicine, 1078 Budapest, Hungary; 2 HUN-REN-UVMB Climate Change: New Blood-sucking Parasites and Vector-borne Pathogens Research Group, 1078 Budapest, Hungary; 3 Centre for Bioinformatics, University of Veterinary Medicine, 1078 Budapest, Hungary; 4 Institute for Animal Breeding, Nutrition and Laboratory Animal Science, University of Veterinary Medicine, 1078 Budapest, Hungary; 5 Czikkhalas Halastavai Ltd., 7067 Varsád, Hungary; 6 Department of Exotic Animal and Wildlife Medicine, University of Veterinary Medicine Budapest, 1078 Budapest, Hungary; 7 HUN-REN Veterinary Medical Research Institute, 1143 Budapest, Hungary; 8 Department and Clinic of Surgery and Ophthalmology, University of Veterinary Medicine, 1078 Budapest, Hungary; 9 Department of Physics of Complex Systems, Eötvös Loránd University, 1117 Budapest, Hungary

**Keywords:** Argulus, Fish lice, Genomics

## Abstract

**Background:**

Species belonging to the genus *Argulus* are globally distributed fish parasites. Their veterinary significance primarily lies in their disruptive presence and their role as mechanical vectors. Although *Argulus japonicus* Thiele, 1900 is a widely distributed representative of this genus that feeds on freshwater fish, only *Argulus foliaceus* (Linnaeus, 1758) had previously been reported in Hungary. The aim of this study was to investigate the fish louse fauna in a local common carp (*Cyprinus carpio* Linnaeus, 1758) population. To the best of our knowledge, this is the first study to report the occurrence of *A. japonicus* in Hungary.

**Methods and results:**

Our detailed molecular analyses, including the complete mitochondrial genome, revealed for the first time that the *A. japonicus* specimens found in Hungary differ significantly from their Far Eastern counterparts. Furthermore, cytochrome c oxidase subunit I (*cox1*) sequence analysis—a region known to be stable within the species—showed that while our sequences were nearly identical to those of other European specimens, they differed markedly from the available Asian isolates. The phylogenetic analysis also confirmed this divergence. The European *A. japonicus* sequences form a clearly distinct sister group to the Asian lineages.

**Conclusions:**

In light of these findings, and on the basis of thorough morphological examinations, the authors propose that the specimens found in Hungary represent a new subspecies, *Argulus japonicus* *europaeus* subsp. nov. Keve, 2025.

**Graphical Abstract:**

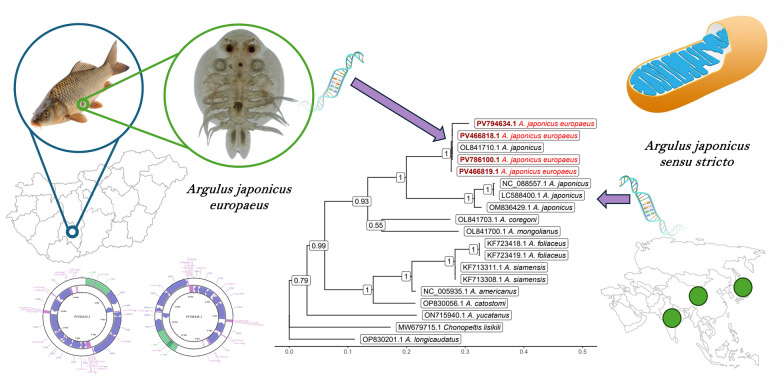

## Background

Fish lice belonging to the Argulidae family are a well-known group of crustacean fish parasites. Only three freshwater species of the family have been confirmed in Europe: *Argulus coregoni* (Thorell, 1864), *Argulus foliaceus* (Linnaeus, 1758) and *Argulus japonicus* Thiele, 1900 (Fryer, 1982) [38]. While fish lice occasionally parasitize amphibians or invertebrates, their primary hosts are fish, [30], including ones of high economic importance, such as cyprinids (*Cyprinus carpio, Chondrostoma* sp., *Squalius cephalus*). The presence of fish lice is disadvantageous at fish farms. [28, 43] This is due to their parasitic nature that causes constant stress to the fish and their role as mechanical vectors for the transmission of various pathogenic agents, such as the spring viremia of carp virus (SVCV) [2]. At the same time, knowledge on the distribution range of *Argulus* species is not complete. The morphological differences between the adults of various *Argulus* species are not always pronounced. Moreover, they have multiple developmental stages, each with somewhat different morphological characteristics [33]. On the other hand, with the rise of molecular identification methods, e.g., species identification based on specific gene sequences, like *cox*1, new doors have opened for taxonomists. Today, the identification of new species should contain morphological and molecular approaches. To date, the scientific literature has documented only one species of fish lice, *A. foliaceus* being present in Hungary [14, 23, 24]; however, on the basis of personal communications, the Hungarian presence of *A. japonicus* was also suspected, although no molecular and/or detailed morphological analyses were documented. The primary objective of this study was to investigate the presence and diversity of pathogens associated with *Argulus* infestation at a fish farm in Hungary, using next-generation sequencing (NGS)-based metagenomic analysis of total DNA extracted from fish lice. Although the initial aim was pathogen detection, the majority of sequencing reads were derived from the parasite itself, enabling the reconstruction of its mitochondrial genome and providing an opportunity to assess its taxonomic identity. Unexpectedly, the genomic data indicated that the specimens were not *Argulus japonicus* sensu stricto (or *A. foliaceus*), as initially presumed. This finding prompted a detailed morphological re-examination of all remaining specimens, complemented by the amplification and sequencing of the cox1 gene from three individuals. These analyses confirmed that the recovered mitogenomes originated from a single *Argulus* subspecies present in the sample collection.

## Results

**Fig. 1:**
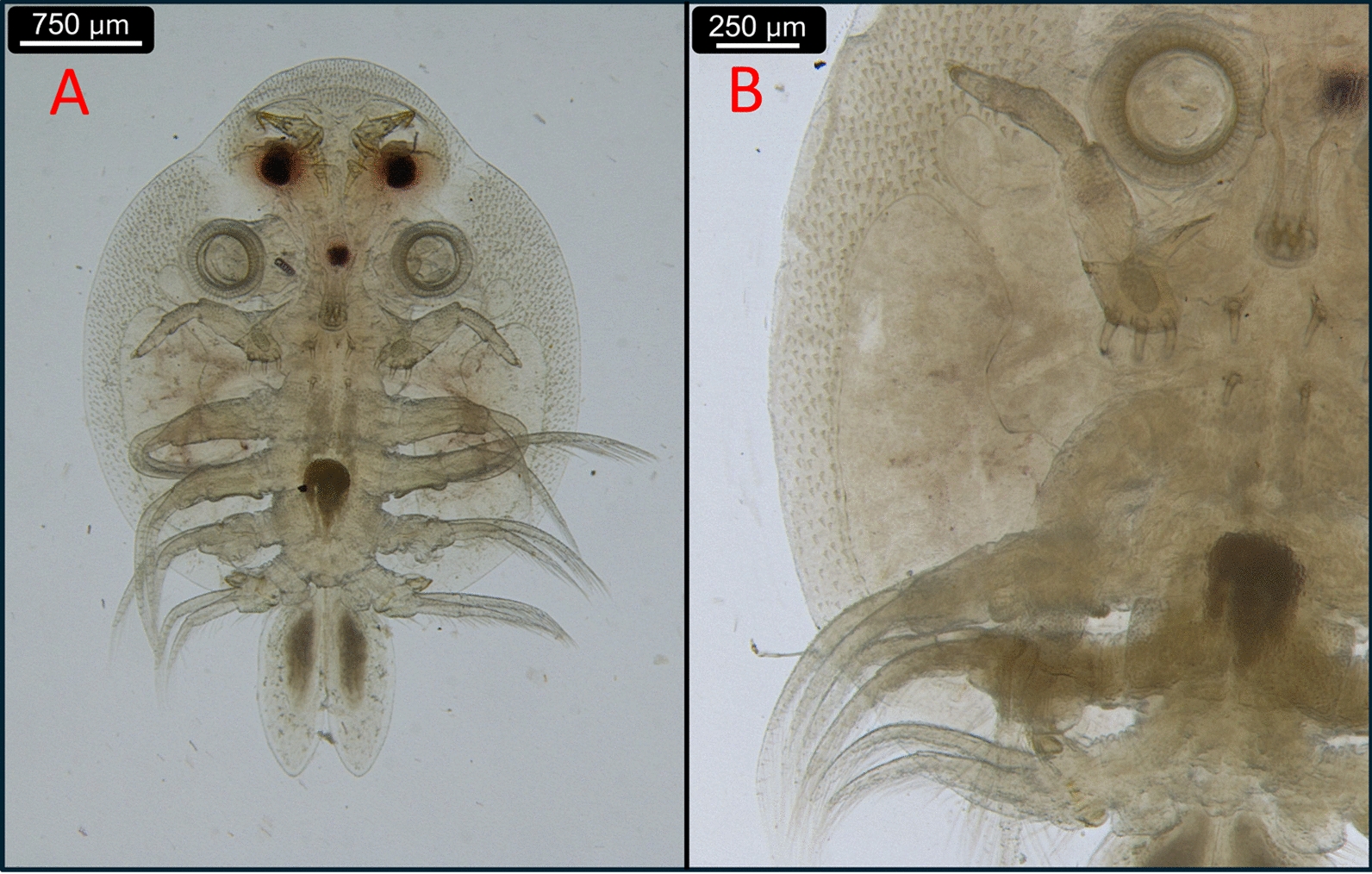
**A**) Ventral aspect of an *A. japonicus* *europaeus* subsp. nov. male;** B**) The respiratory areas of the same specimen

**Fig. 2:**
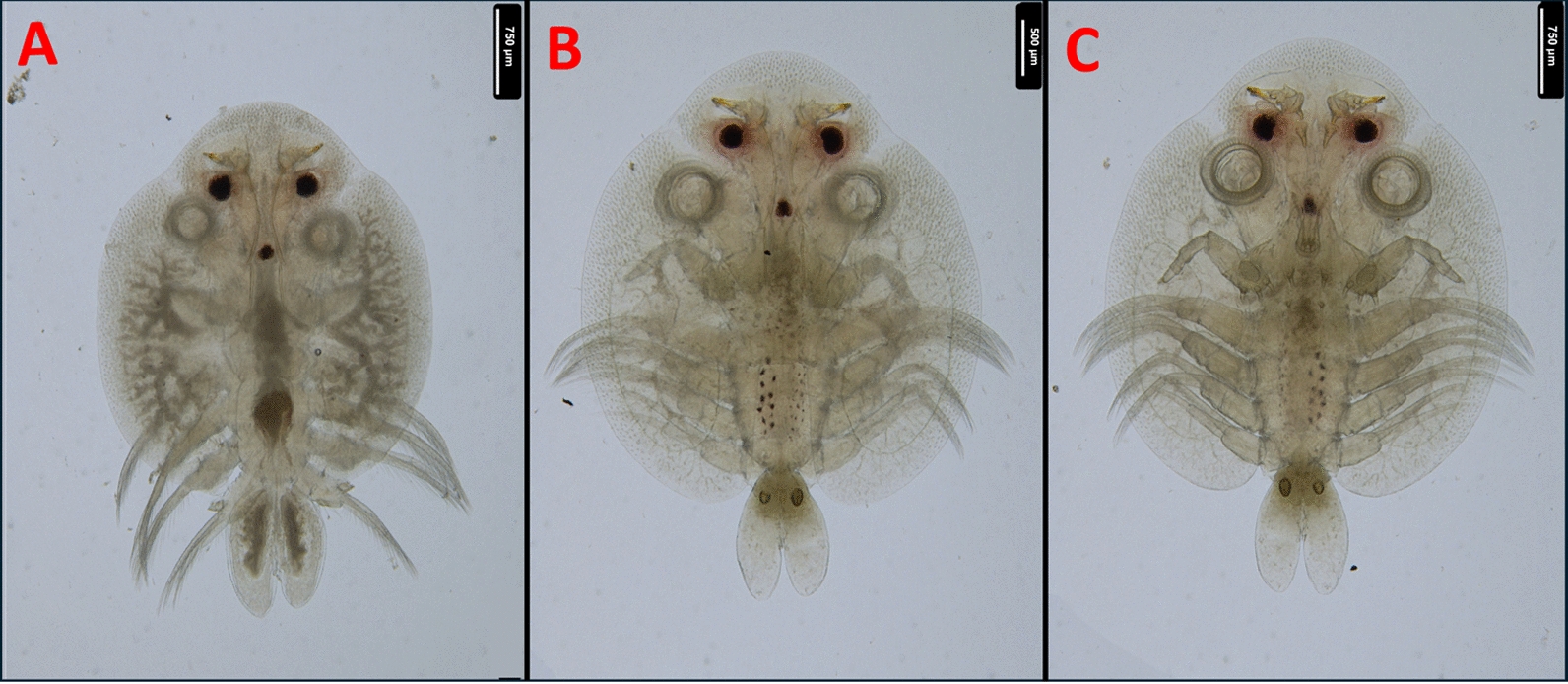
**A**) Dorsal aspect of a male (this specimen is different from the one on Fig. 1);** B**) Dorsal aspect of a female;** C**) Ventral aspect of a female *A. japonicus* *europaeus* subsp. nov

**Fig. 3:**
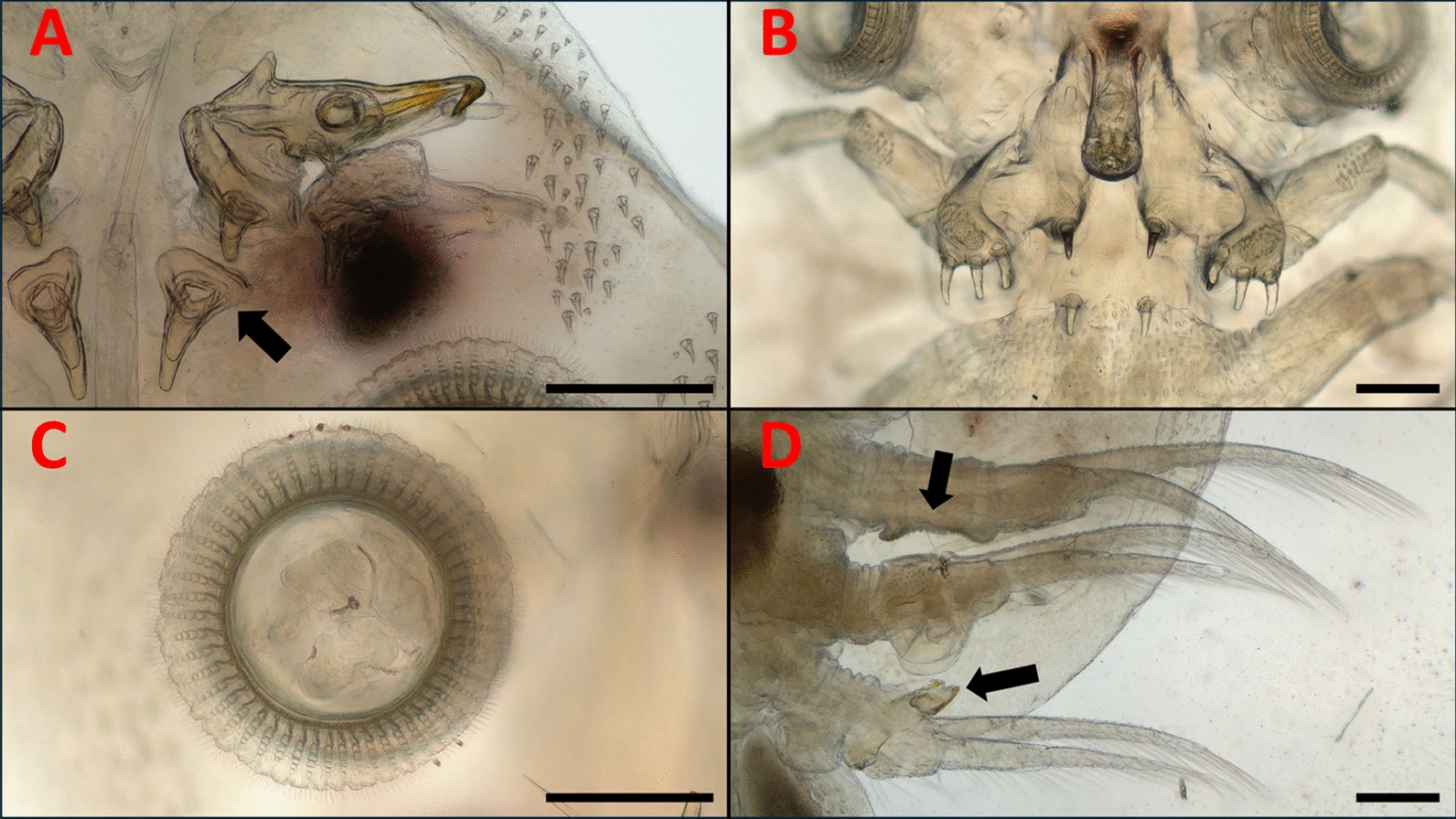
The relevant morphological structures of an *A. japonicus* *europaeus* subsp. nov. male specimen.** A**) Antennae and stylet (arrow: widened basis of the post antennal spine);** B**) Secondary maxillae and mouth tube (50x magnitude);** C**) Sucker and its supporting rods;** D**) The 2nd, 3rd, and 4th legs (arrows: clasping apparatus on legs 2 and 4, characteristic to *A. japonicus*. Scales: 250 μm

**Fig. 4:**
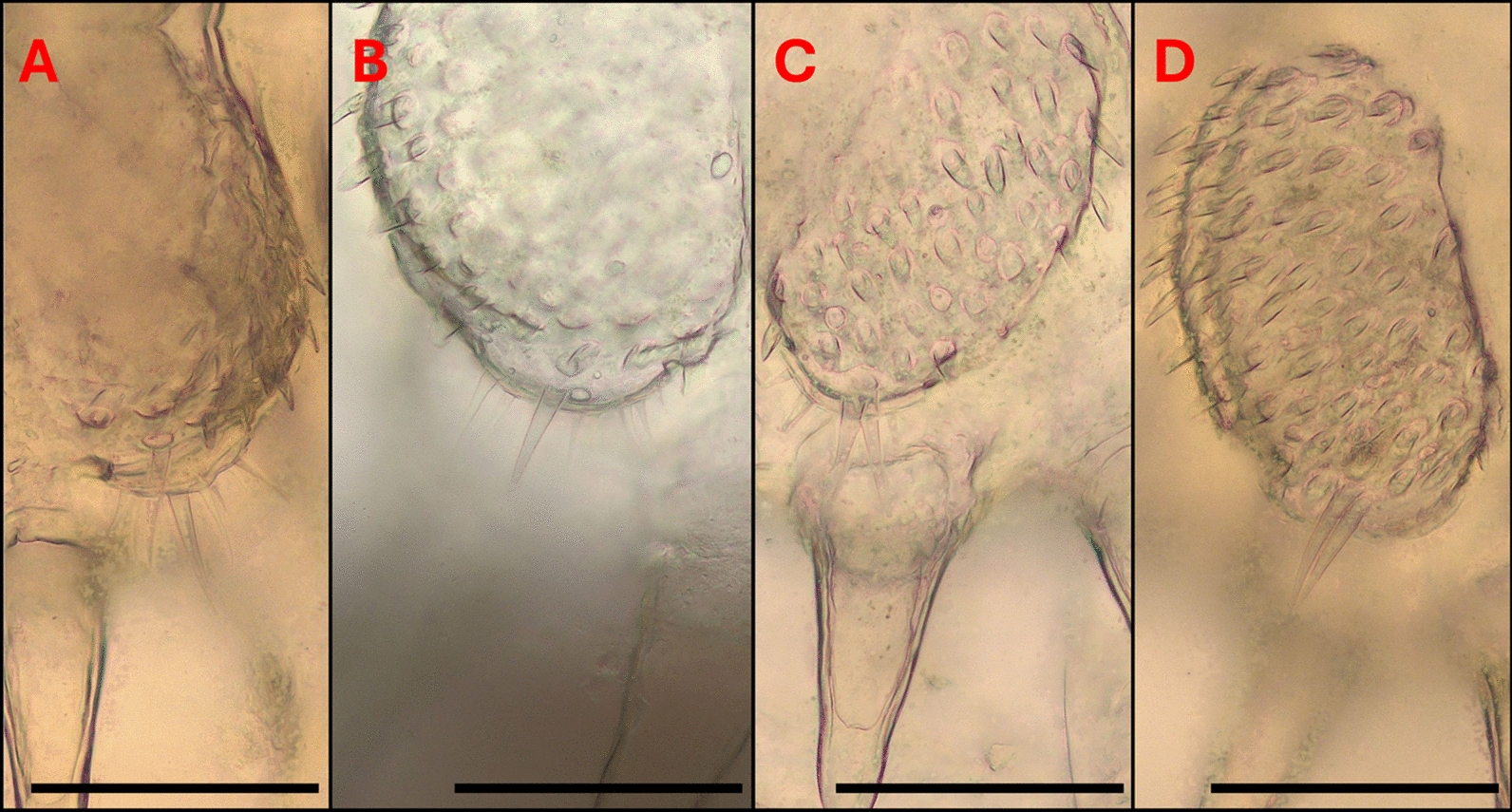
Setae on the margin of the secondary maxillae of females (**A**,** B**) and males (**C**,** D**) scales: 100 μm

**Fig. 5:**
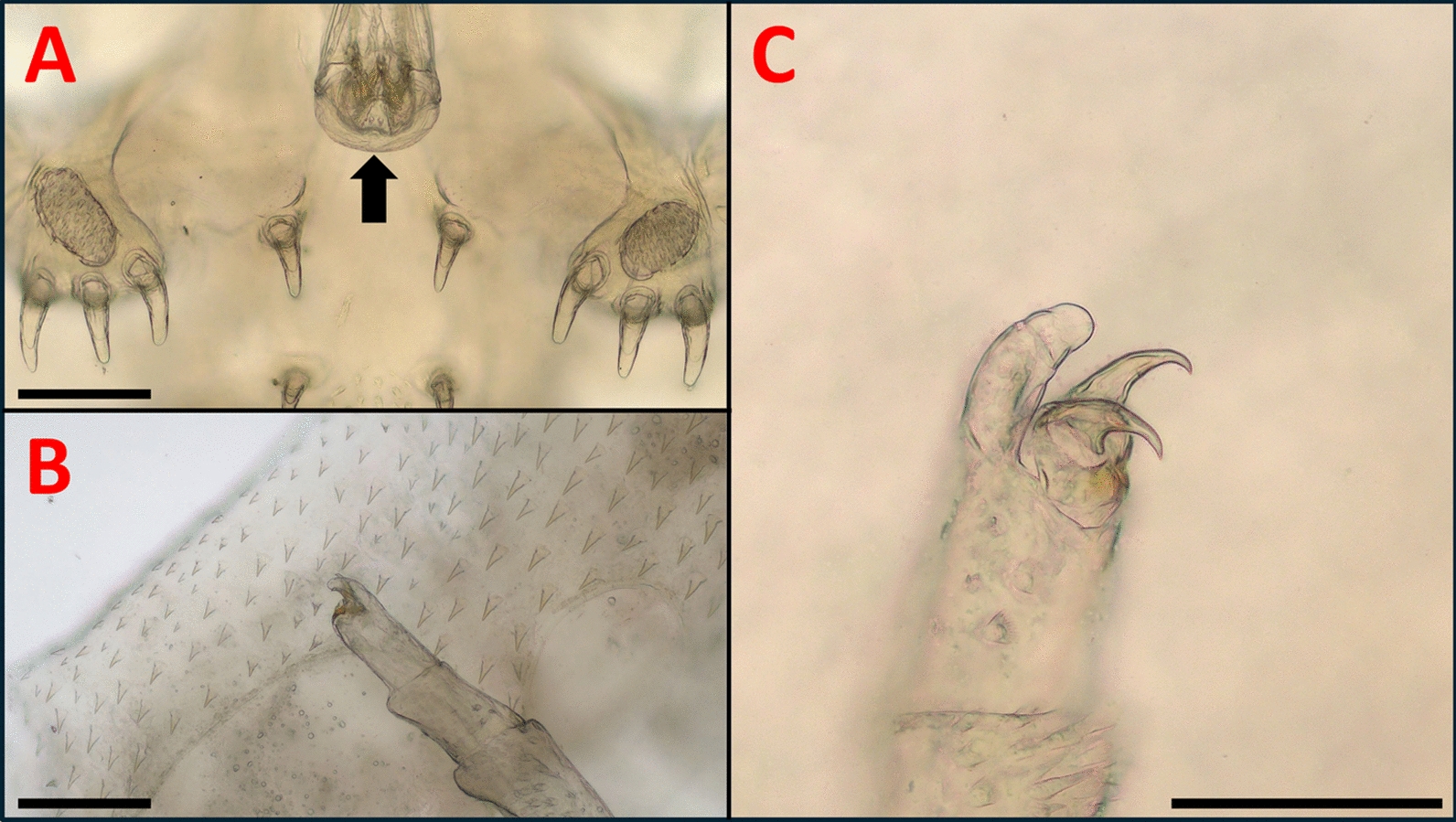
**A**) Labium (arrow) and secondary maxillae of a male;** B**) Final segment of the secondary maxilla of a female;** C**) Final segment of the secondary maxilla of a male. Scales: 100 μm

**Fig. 6:**
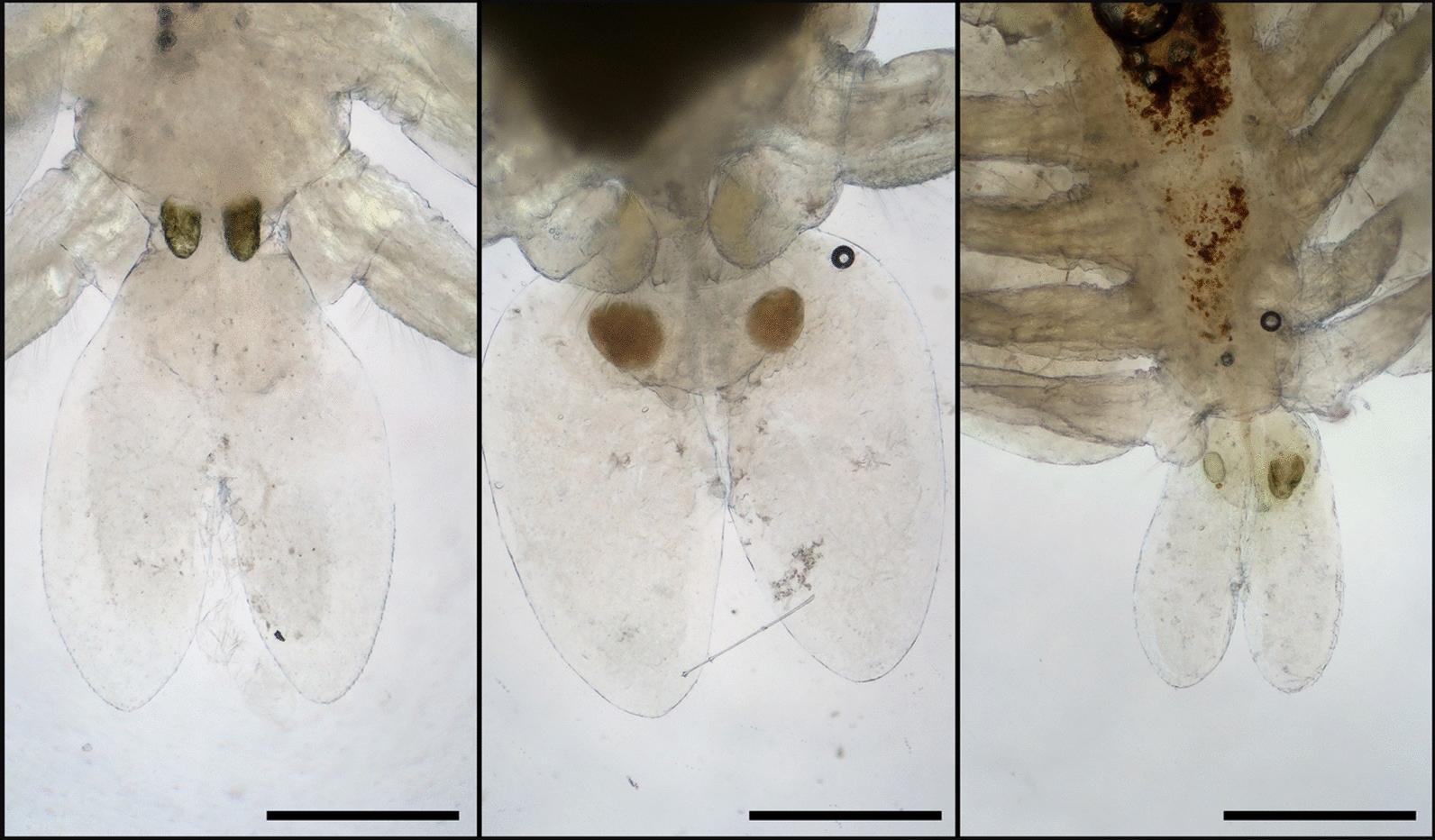
Abdominal lobes of three different female specimens. Scales: 500 μm

According to our morphological analyses, all the fish lice we found belong to the *A. japonicus* species: The coxae with the clasping apparatus of the second, third, and fourth legs of the males have the same appearance as described in the work of Fryer [9]. (Fig. 1/A, Fig. 3/D). Other works, such as those of Rushton-Mellor and Boxshall, and Shoes et al. [33, 38] also underline, that the most reliable morphological attributes based on which *A. japonicus* and *A. foliaceus* can be distinguished are the different accessory copulatory structures on legs 2, 3, and 4 of the males.

In the case of both males and females, the abdominal lobes are acutely rounded, as described in the work of Fryer [9], although, according to our observations, their general shapes in females were variable (Fig. 6). The larger respiratory areas are reniform and posterior to the smaller respiratory areas (Fig. 1/B). On the antennae, distinct, sharp terminal, anterior, medial, posterior, and post-antennal spines are visibe (Fig. 3/A).

Basal plates are present on the second maxillae. The coxal spines of the secondary maxillae are long and finger-like (Fig. 3/B). Scales are present on the labium, although rather subtle (Fig. 5/A).

The basal sclerites of the supporting rods of the suckers are elongated (number of supporting rods: 52–53 in males and females, with 5–7 sclerites in each rod. (Fig. 3/C). This is more or less in line with the observations of Nagasawa [25] (50–52 rods in a female) and those of Wadeh et al. [41] (45–53 rods). The hooks on the final segments of the second maxillae of both females and males are similar to the figures in the work of Nagasawa [25] (Fig. 5/B,C).

Relevant differences were observed in some specimens, compared with the works of Nagasawa [25] and Rushton-Mellor [34]: the post antennal spines on our specimens have widened basis in contrast to the specimens described in the aforementioned works (Fig. 3/A). On the margin of the basal plate of the secondary maxilla, we observed two long setae along with several medium-long setae (instead of just two long setae, as in the aforementioned works) (Fig. 4) A/B. On the other hand, among males, some specimens had only two long setae, similarly to the descriptions of Rushton-Mellor and Nagasawa [25, 34]. Other males possessed multiple setae, just as the females (Fig. 4C/D). However, in his work, Bauer [3] also depicted these medium-length setae in his illustration of an *A. japonicus* male. In the latter illustration, the post antennal spines are absent.

The abdominal incision of the female is less than half of the abdominal length in contrast to the description of Fryer [9] (Fig. 6). The total body length of females was in the range of *A. japonicus* 3.12–6.27 mm (mea*n* = 4.67, *n* = 12), while the length of males was between 3.81–5.49 mm (mea*n* = 4.55, *n* = 9). Even though these measurements differ from the sizes reported by Wadeh et al. [41] (Fig. 2/A,B,C), a comparison should not be made, since these crustaceans were potentially in different life stages. The most relevant morphological differences are summarized in Table 1. For this table, only the differences compared with the reports of Rushton-Mellor [34] and Nagasawa [25] were considered, as -following the results of Wadeh et al. [42] - priority was given to African and Asian descriptions. For this reason, the previously mentioned similarity between the spines on the margin of the basal plate of the secondary maxilla and the description by Bauer [3] was not depicted, as the referenced specimen originated from Russia.

We assembled an MT-derived contig from the Illumina sequencing, while a long read from the ONT sequencing was found to be MT-derived.

**Table 1:** Relevant morphological differences between *Argulus japonicus* sensu stricto and *Argulus japonicus europaeus*

Character	*Argulus japonicus* sensu stricto	*Argulus japonicus europaeus*
Basis of post antennal spine	Tapering more or less evenly [25, 34]	Has a pronouncedly widened basis
Spines on the margin of the basal plate of the secondary maxilla	Both sexes: only two long spines are present [25, 34]	Males: only two long spines, or two long spines along with several medium-length spines are present. Females: Two long spines along with several medium-length spines are present

The length of the assembled MT-sequence (GenBank: PV786100.1) of Arg_hun1 sample reached 15004 bp. Detailed annotation of this sequence can be found in Table 2 and (Fig. 8). Since only *A. foliaceus* has been scientifically proven to be present in Hungary, the first comparison aimed at this species as the reference sequence for our MT sequence. However, no complete *A. foliaceus* sequences are available in the NCBI NT database, only shorter fragments of it. Consequently, the basis of the comparison relied on common, incomplete MT-sequences that overlapped in multiple *Argulus* species, encompassing the 10810–11442 region of our assembled MT-sequence. This segment showed the highest sequential similarity to *A. japonicus* (OL841710.1) *cox*1 (identity: 565/565 (100%), gaps: 0/565 (0%)), escorted by the following hits: *A. mongolianus* (OL841700.1, identity: 467/574 (81%), gaps: 0/574 (0%)), *A. coregoni* (OL841703.1, identity: 461/565 (82%), gaps: 0/565 (0%)), *A. americanus* (NC_005935.1, identity: 494/633 (78%), gaps: 0/633 (0%)), *A. siamensis* (KF713308.1, identity: 486/633 (77%), gaps: 0/633 (0%)), *A. foliaceus* (KF723419.1, identity: 478/633 (76%), gaps: 0/633 (0%)), and *A. yucatanus* (ON715940.1, identity: 411/528 (78%), gaps: 0/528 (0%)). The complete MT-sequence was associated with higher sequential identity to *A. japonicus* (LC588400.1: coverage: 98%, identity: 84.82%; NC_088557.1: coverage: 98%, identity: 84.68%) than with *A. americanus* (NC_005935.1: coverage: 90%, identity: 76.58%).

The 619 bp length *cox*1 sequences of one male and two females were received (accession number: PV466818) after Sanger sequencing were 100% identical to European *A. japonicus* sequences: OL841708.1 - OL841710.1 (565/565 (100%), gaps: 0/565 (0%)) as well. These sequences showed a low sequential similarity ($\tilde{8}3\%$) to Japanese and Chinese *A. japonicus* sequences (LC588400.1 and NC_005935.1; 513 and 512/618 bp identity, respectively, with 0 gaps). The one male specimen (accession number: PV466819) differed from the two females (accession number: PV466818) by only one nucleotide (618/619 bp, 99.84%)

The length of the MT-sequence (GenBank: PV794634.1) of Arg_hun2 sample reached 16748 bp. Detailed annotation of this sequence can be found in Table 2 and Fig. 8. The same segment as above showed the highest sequential similarity to *A. japonicus* (OL841710.1) *cox*1 (identity: 545/569 (96%), gaps: 12/569 (2%)), escorted by the following hits: *A. mongolianus* (OL841700.1, identity: 456/578 (79%), gaps: 14/578 (2%)), *A. coregoni* (OL841703.1, identity: 447/568 (79%), gaps: 5/568 (1%)), *A. americanus* (NC_005935.1, identity: 484/639 (76%), gaps: 15/639 (2%)), *A. longicaudatus* (OP830201.1, identity: 480/634 (76%), gaps: 13/634 (2%)), *A. catostomi* (OP830056.1, identity: 482/637 (76%), gaps: 13/637 (2%)), *A. siamensis* (KF713311.1, identity: 476/638 (75%), gaps: 13/638 (2%)), *A. foliaceus* (KF723419.1, identity: 468/638 (73%), gaps: 13/633 (2%)), and *A. yucatanus* (ON715940.1, identity: 402/533 (75%), gaps: 10/533 (1%)). The complete MT-sequence was associated with higher sequential identity to *A. japonicus* (LC588400.1: coverage: 87%, identity: 83.71%; NC_088557.1: coverage: 87%, identity: 83.49%) than with *A. americanus* (NC_005935.1: coverage: 79%, identity: 75.26%).

**Fig. 7:**
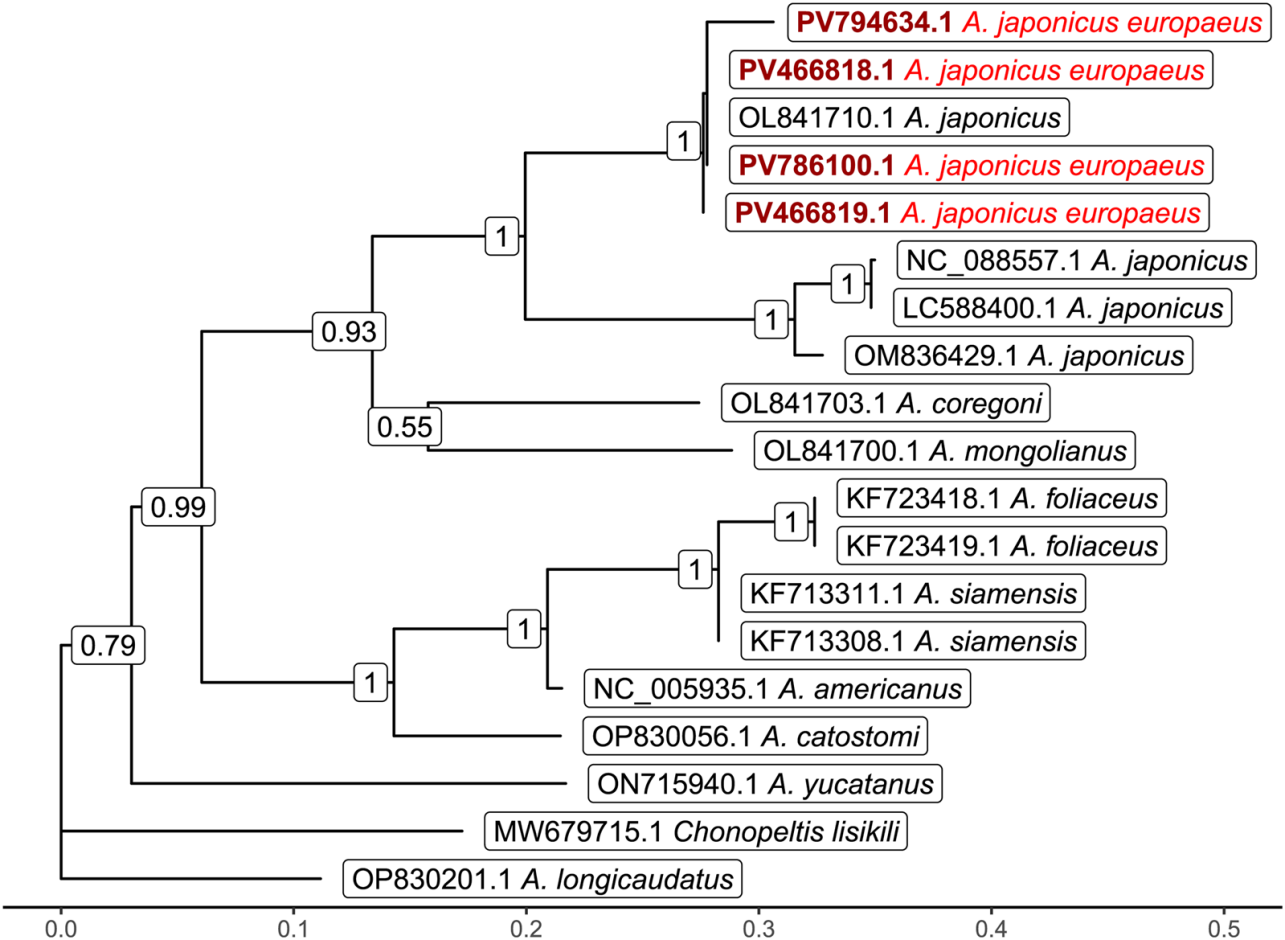
Gene tree based on the overlapping segment of *cox*1. Outgroup: *Chonopeltis lisikili* (MW679715.1). Numbers at branches indicate bootstrap support levels (100 replicates). Sequences from this study are shown in red font

The BLAST alignment of the PV786100.1 sequence on PV794634.1 gives 100% coverage and 97.97 % sequential identity. Figure 7 shows the gene-tree based on the above-mentioned overlapping *cox*1 sequences with the best substitution model, TVM. On this tree, it is visible that *A. japonicus* samples from Asia (China, Japan, and India) form a highly distinct sister group compared with European samples (Hungary and England), with strong statistical support (1).

On the basis of the morphological, and genetic differences compared with the (Asian) *A. japonicus* samples, it is assertable that the Hungarian *A. japonicus*, as well as some other European specimens reported before belong to a new subspecies: *Argulus japonicus* *europaeus* subsp. nov. Keve, 2025. The holotype (male specimen, collected by Adrienn Gréta Tóth and Norbert Solymosi on 07/10/2024, Hungary) is deposited in the Department of Parasitology and Zoology, University of Veterinary Medicine, Budapest (accession number: UNIVET-PAR-KG001.) ZooBank registration: To comply with the regulations set out in article 8.5 of the amended 2012 version of the International Code of Zoological Nomenclature (ICZN) [27], details of the new subspecies have been submitted to ZooBank. The Life Science Identifier (LSID) of the article is LSIDurn:lsid:zoobank.org:pub:27F531B8–87D0–4CE3-A98C-6D526E6D7795. The LSID for the new name *Argulus japonicus* *europaeus* Keve, 2025 is LSIDurn:lsid:zoobank.org:act:4D58B113–04B5–472A-AA8E-A015F5AA8449. The sequences obtained in the current study were deposited in the GenBank database and are available under the following accession numbers: PV466818-PV466819, PV786100, PV794634.

**Table 2:** Annotation of the mitogenome of the two sequenced samples. The sequential identity of the detected 37 features with the reference genome (LC588400.1) features was calculated by Needleman–Wunsch global alignment

Gene name	Type	Gene product	PV786100.1					PV794634.1				
			Start	End	Length	Strand	Identity	Start	End	Length	Strand	Identity
					bp		%			bp		%
ATP6	CDS	ATP synthase F0 subunit 6	8342	9002	660	−	81.4	1201	1860	659	−	79.5
ATP8	CDS	ATP synthase F0 subunit 8	8996	9151	155	−	73.9					
COX1	CDS	cytochrome c oxidase subunit I	9954	>11490	1536	−	84.5	2805	4362	1557	−	82.4
COX2	CDS	cytochrome c oxidase subunit II	9213	9885	672	−	85.1	2051	2742	691	−	83.8
COX3	CDS	cytochrome c oxidase subunit III	7558	8341	783	−	87.4	419	1202	783	−	87.2
CYB	CDS	cytochrome b	12885	14027	1142	−	81.5	5738	6866	1128	−	81.2
NAD1	CDS	NADH dehydrogenase subunit 1	1983	2891	908	+	86.0	11525	12454	929	+	85.2
NAD2	CDS	NADH dehydrogenase subunit 2	11700	12662	962	−	78.3	4660	5543	883	−	76.2
NAD3	CDS	NADH dehydrogenase subunit 3	7155	7496	341	−	76.3	8	358	350	−	71.4
NAD4	CDS	NADH dehydrogenase subunit 4	3741	5016	1275	+	83.2	13444	14564	1120	+	81.4
NAD4L	CDS	NADH dehydrogenase subunit 4 L	3445	3747	302	+	85.6	13002	13302	300	+	85.6
NAD5	CDS	NADH dehydrogenase subunit 5	5078	6719	1641	+	79.7	14626	16269	1643	+	78.4
NAD6	CDS	NADH dehydrogenase subunit 6	2908	3354	446	−	76.2	12462	12925	463	−	75.6
l-rRNA	rRNA	16 S ribosomal RNA	934	1982	1048	+	89.9					
s-rRNA	rRNA	12 S ribosomal RNA	227	866	639	+	94.2	9708	10411	703	+	92.9
trnA(tgc)	tRNA	tRNA-Ala	7097	7156	59	−	98.4	16640	16702	62	−	93.7
trnC(gca)	tRNA	tRNA-Cys	11571	11631	60	+	96.7	4426	4487	61	+	93.4
trnD(gtc)	tRNA	tRNA-Asp	9153	9212	59	−	95.0	2004	2065	61	−	93.4
trnE(ttc)	tRNA	tRNA-Glu	6781	6844	63	−	90.6	16324	16387	63	−	89.1
trnF(gaa)	tRNA	tRNA-Phe	6720	6780	60	+	93.4	16264	16326	62	+	93.7
trnG(tcc)	tRNA	tRNA-Gly	7497	7557	60	−	91.8	358	419	61	−	91.8
trnH(gtg)	tRNA	tRNA-His	5017	5077	60	+	93.4	14564	14626	62	+	87.1
trnI(gat)	tRNA	tRNA-Ile	14104	14164	60	−	98.4	6960	7017	57	−	90.2
trnK(ttt)	tRNA	tRNA-Lys	6971	7040	69	−	90.0	16515	16584	69	−	89.9
trnL1(tag)	tRNA	tRNA-Leu	12806	12863	57	+	89.7	5654	5717	63	+	87.3
trnL2(taa)	tRNA	tRNA-Leu	9886	9953	67	−	98.5	2743	2810	67	−	98.5
trnM(cat)	tRNA	tRNA-Met	12678	12741	63	−	98.4	5531	5595	64	−	98.4
trnN(gtt)	tRNA	tRNA-Asn	6912	6970	58	−	93.2	16454	16515	61	−	93.2
trnP(tgg)	tRNA	RNA-Pro	3355	3415	60	+	93.4	12910	12970	60	+	90.2
trnQ(ttg)	tRNA	tRNA-Gln	11505	11572	67	+	97.1	4361	4428	67	+	95.6
trnR(tcg)	tRNA	tRNA-Arg	7039	7098	59	−	93.4	16584	16640	56	−	93.0
trnS1(tct)	tRNA	tRNA-Ser	6844	6911	67	−	94.1	16386	16455	69	-	91.3
trnS2(tga)	tRNA	tRNA-Ser	14603	14660	57	−	95.0	7439	7497	58	−	95.0
trnT(tgt)	tRNA	tRNA-Thr	14493	14552	59	−	96.7					
trnV(tac)	tRNA	tRNA-Val	867	929	62	+	93.8	10409	10472	63	+	93.8
trnW(tca)	tRNA	tRNA-Trp	14042	14104	62	−	92.1	6898	6961	63	−	92.1
trnY(gta)	tRNA	tRNA-Tyr	11636	11696	60	+	93.4	4494	4553	59	+	88.5

**Fig. 8:**
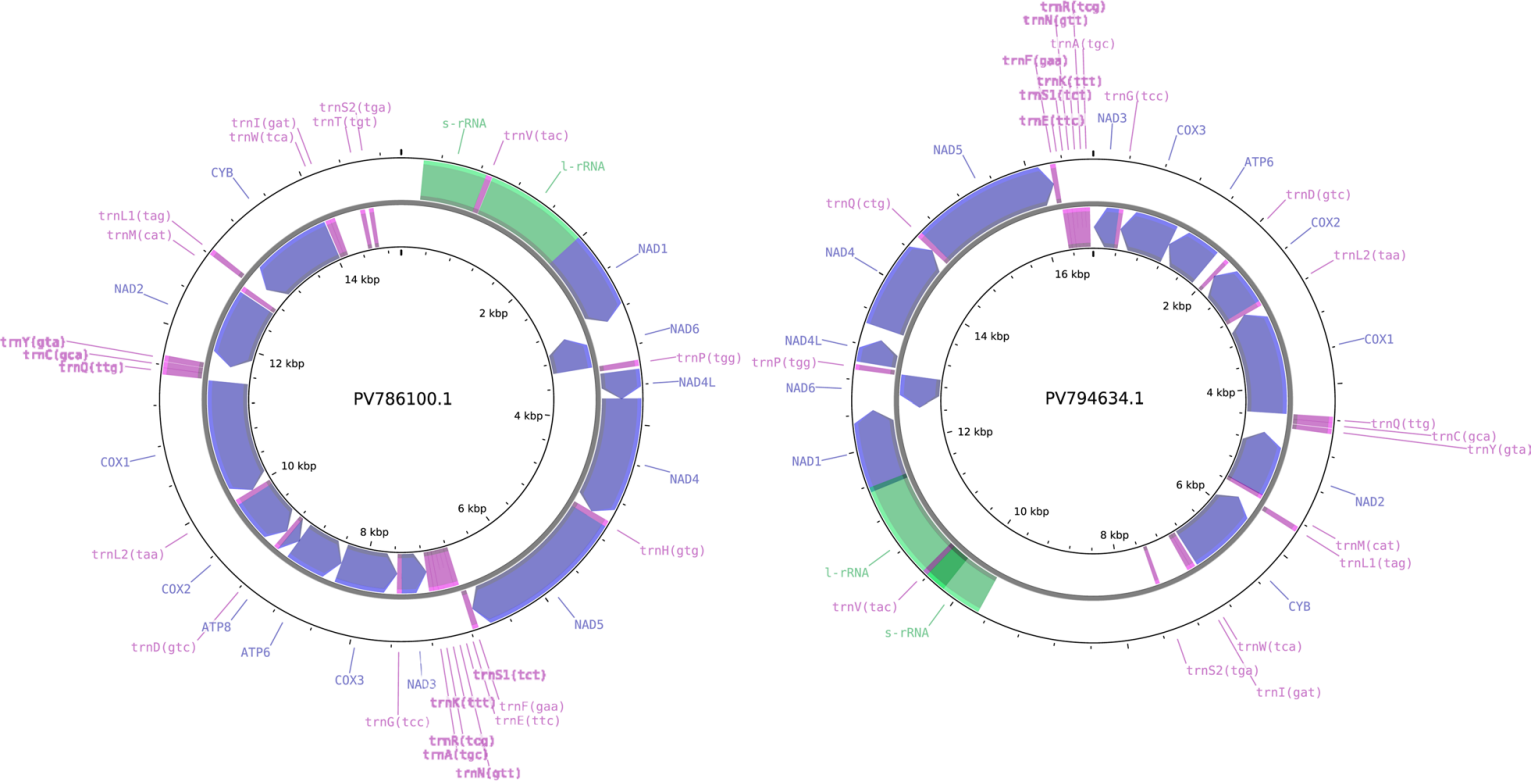
Mitogenome annotations. In the PV786100.1 assembly 37, while in the PV794634.1 contig 34 features (CDS, rRNA, tRNA) were detected

## Methods

### Sampling and morphology

The sample collection was conducted at a fish farm in the southern Transdanubian region of Hungary (Tolna County, Hungary; coordinates: 46.53, 18.50). The first sample collection was performed on 05/04/2024. During this sample collection, fish lice were collected from one carp. The second set of samples was gathered on 07/10/2024, from a different pond of the same system. This time, fish lice samples were randomly collected from more than three different carps. In all cases, the fish lice were removed during sampling for veterinary purposes. The samples were stored at $-20^{\circ }$C.

For morphological identification, *Argulus* specimens (nine males and twelve females) were put into isotonic saline and were observed with stereo (Leica M205C) and with light microscopes (Leica DM2000) (Leica Microsystems, Wetzlar, Germany). The morphological identification of *A. japonicus* was based on morphological keys and on relevant articles [3, 9, 25, 38, 40, 41]. Photography for Fig. 1 and Fig. 2 were made with a stereo microscope and compatible camera Leica DMC4500, and were compiled and measured with LasX (Leica application suite X) program (Leica Microsystems, Wetzlar, Germany). Photography for Fig. 3, Fig. 4, Fig. 5, Fig. 6 were made with light microscope and compatible camera Leica FLEXACAM C1 (Leica Microsystems, Wetzlar, Germany) and were compiled with CombineZP program [11]. For measurements, both microscopes were calibrated with the same objective micrometer. Scales were added or adjusted manually.

### Sequencing

DNA purification from the samples was performed in triplicates, and the resulting total DNA extracts were pooled together. DNA extraction for Illumina and Nanopore sequencing were carried out using ZymoBIOMICS DNA/RNA miniprep kits (R2002, Zymo Research, Irvine, USA). For Illumina sequencing, we used a specimen from the first sample collection, while four subjects for nanopore sequencing were selected from the second sample collection.

For efficient sample lysis, bead homogenization was performed using a Vortex-Genie 2 with a bead size of 0.1 mm and a homogenization time of 15 min at maximum speed. After that, the Zymo Research kit’s DNA purification protocol was followed. Total DNA qualities were assessed with an Agilent 2200 TapeStation instrument (Agilent Technologies, Santa Clara, USA), and DNA quantities were measured using a Qubit Flex Fluorometer (Thermo Fisher Scientific, Waltham, MA, USA). We closely followed all manufacturer recommendations when preparing sequencing libraries for Illumina sequencing platform (Illumina Inc., San Diego, USA). Pooled total DNA samples were constructed using the NEBNext Ultra II Library Prep Kit (NEB, Ipswich, MA, USA). Paired-end shotgun metagenome sequencing was performed on a NextSeq 550 (Illumina, San Diego, CA, USA) sequencer using the NextSeq High Output Kit v2 sequencing reagent kit. Primary data analysis (i.e., base-calling) was performed using “bcl2fastq” software (version 2.17.1.14, Illumina).

For Sanger sequencing, the specimens (two males, including the one in Fig. 1, and two females from the second sample collection) were disinfected on their surface with sequential washing for 15 s in detergent, tap water, and distilled water. For the DNA extraction, legs and pieces of the carapace were cut off and used. DNA was extracted with the QIAamp DNA Mini Kit (QIAGEN, Hilden, Germany) according to the manufacturer’s instructions, including an overnight digestion in tissue lysis buffer and Proteinase-K at 56 °C. Extraction controls (tissue lysis buffer) were also processed with the *Argulus* samples to monitor cross-contamination.

The *cox*1 gene was chosen as the first target for molecular analysis. The PCR was modified from Folmer et al. [8] and amplifies an approx. 710-bp-long fragment of the gene. The primer LCO1490 (5’-GGT CAA CAA ATC ATA AAG ATA TTG G-3’) were used in a reaction volume of 25 µl, containing 1 U (stock 5 U/ µl) HotStarTaq Plus DNA Polymerase, 2.5 µl 10$\times$ CoralLoad Reaction buffer (including 15 mM MgCl_2_), 0.5 µl PCR nucleotide Mix (stock 10 mM), 0.5 µl of each primer (stock 50 µM), 15.8 µl dd H_2_O and 5 µl template DNA. For amplification, an initial denaturation step at $95^{\circ }$C for 5 min was followed by 40 cycles of denaturation at $94^{\circ }$C for 40 s, annealing at $48^{\circ }$C for 1 min and extension at $72^{\circ }$C for 1 min. Final extension was performed at $72^{\circ }$C for 10 min.

In all PCRs nontemplate reaction mixture served as negative control. Extraction controls and negative controls remained PCR negative in all tests. Purification and Sanger sequencing of the PCR products were done by Biomi Ltd. (Gödöllő, Hungary).

### Bioinformatic analysis

For raw Illumina sequenced data, the quality-based filtering and trimming of the raw short reads were performed by TrimGalore (v.0.6.6, https://github.com/FelixKrueger/TrimGalore), setting 20 as the quality threshold. Only reads longer than 50 bp were retained. Using default settings, the cleaned reads were assembled to contigs by MEGAHIT (v1.2.9) [20].

The basecalling was performed using dorado (https://github.com/nanoporetech/dorado, v0.9.0) with model dna_r10.4.1_e8.2_400bps_sup@v5.0.0, based on the POD5 files generated by the ONT Mk1C sequencer. The raw reads were adapter-trimmed and quality-based filtered by Porechop (v0.2.4, https://github.com/rrwick/Porechop) and Nanofilt (v2.6.0, minimal Q=7, length=50) [7], respectively.

The assembled contigs and the ONT long reads were taxon classified by Kraken2 (v2.1.4) [44] using the NCBI Core NT database (created: 12/28/2024). The *Argulus* hits were analysed by BLAST [5] on NCBI Core NT database (accessed: 01/03/2025). The MitoZ [21] annotated mitogenomes were visualized by Proksee [10]. The identity of detected mitogenome features was analyzed in the R-environment [31] by the Needleman–Wunsch global alignment algorithm of package pwalign [1]. Phylogenetic analysis was performed based on the COX1 overlapping region. The gene-tree was constructed [45] on the basis of multiple sequence alignment by MAFFT (v7.490) [16]. The best substitution model was selected by functions of phangorn (v2.11.1) package [36] based on the Bayesian information criterion. The generated neighbor-joining tree was optimized by the maximum likelihood method. Bootstrap values were produced by 100 iterations. All data processing and plotting were done in the R-environment [31].

## Discussion

*Argulus japonicus* was originally described in China. Besides its Asian distribution including Bangladesh, China, India, Japan, Syria, and Turkey [15, 18, 26, 35, 41, 42], its global spread is also reported. Its appearance is recorded in Africa, Australia, Europe, North America, and South America [4, 6, 12, 19, 41, 42]. However, its precise distribution range is less studied and several countries lack information on the presence of the parasite. In Europe, *A. japonicus* was described in Bosnia and Herzegovina, Croatia, Montenegro, France, Germany, Greece, Italy, the Netherlands, Norway, Poland, the UK, Serbia, Slovakia, and Spain [13, 17, 29, 32, 37–39]. Although many of these reports are related to fish trading and imported ornamental fish, several findings are derived from fish farms or wild waters.

To the best of our knowledge, this is the first report confirming the presence of *A. japonicus* in Hungary and the first study to provide the complete mitochondrial genome of a European *A. japonicus* specimen. However, this 15,004 bp sequence (PV786100) exhibits low similarity (85%) to previously reported Japanese (LC588400: identity 9627/11313 [85%], gaps 99/11313 [0%]) and Chinese (NC_088557: identity 9620/11324 [85%], gaps 117/11324 [1%]) sequences. In addition, on the basis of their morphology and *cox1* sequences, these fish-lice we found are identical to other *A. japonicus* found in Europe, and differ from *A. foliaceus*, a species that is known to be present in Hungary (Fig. 7). This is despite the fact, that the *cox*1 gene is reported to be relatively stable in the case of the species *A. japonicus* from Asia and Africa (variations are between 0.0–1.9%) [42], and also suitable for species differentiation [22].

Notably, our phylogenetic analysis based on the *cox*1 gene reveals that *A. japonicus* samples from Asia (China, Japan, and India) form a highly distinct sister group compared with European samples (Hungary and England), with strong statistical support (1) (Fig. 7). This finding raises an important question: Are the *A. japonicus* specimens reported in various European countries truly the same species as the *A. japonicus* commonly found parasitizing freshwater fish in Asia? In this study, we highlight the morphological similarities between these lineages while also addressing their genetic diversity. On the basis of our findings, we strongly suspect that *A. japonicus* represents a species complex with distinct lineages in Europe and Asia, rather than a single species. (Fig. 8)

## Conclusions

To determine the extent of this ectoparasite’s presence, comprehensive studies would be required to evaluate its distribution and frequency across Hungary. Additionally, given that no data is available on the simultaneous occurrence of the fish lice species, further sample collection and co-occurrence studies of *A. foliaceus* and *A. japonicus* could enhance our understanding of the infestation risk and possible interbreeding. Nevertheless, on the basis of the slight morphological and distinct molecular differences, we recognize the fish lice discussed in this study as a new subspecies of *A. japonicus*, namely *A. japonicus* *europaeus* subsp. nov. Keve 2025.

## Data Availability

The sequences obtained in the current study were deposited in the GenBank database and are available under the following accession numbers: PV466818-PV466819, PV786100, PV794634.
